# Frequency of antibiotic associated diarrhea caused by *Clostridium difficile* among hospitalized patients in intensive care unit, Kerman, Iran 

**Published:** 2017

**Authors:** Ebrahim Rezazadeh Zarandi, Shahla Mansouri, Nouzar Nakhaee, Farhad Sarafzadeh, Zahra Iranmanesh, Mohammad Moradi

**Affiliations:** 1 *Department of Microbiology and Virology, Faculty of Medicine, Kerman University of Medical Sciences, Kerman, Iran*; 2 *Department of Community Medicine, Faculty of Medicine, Kerman University of Medical Sciences, Kerman, Iran*; 3 *Department of Infectious Diseases, Afzalipour Hospital, Kerman University of Medical Sciences, Kerman, Iran*

**Keywords:** *Clostridium difficile*, Intensive Care Unit, CDAD

## Abstract

**Aim:**

This study evaluated the frequency of C. difficile and CDAD in the ICU of Shahid Bahonhar Hospital, Kerman, Iran.

**Background:**

*Clostridium difficile* (*C. difficile*) is the most important antibiotic associated diarrhea agent in intensive care unit (ICU) patients. Based on its toxin producing ability, C .difficile is divided to toxigenic and non-toxigenic strains.

**Methods:**

A total of 233 diarrheal samples were collected from ICU patients. The samples were cultured on Clostridium difficile medium with 5% defibrinated sheep blood containing cycloserine (500 mg/L), cefoxitin (16 mg/L) and lysozyme (5mg/L). The isolates were confirmed as C. difficile by polymerase chain reaction (PCR) of 16s rRNA gene and the presence of toxins genes (tcdA, tcdB, cdtA and cdtB) was also confirmed. Then, the toxin production of isolates was evaluated using ELISA.

**Results:**

C. difficile was isolated from 49 (21%) out of 233 samples. The total isolates fell into the A-/B-/CDT- (48.97%), A+/B-/CDT- (28%), A+/B+/CDT- (20.4%) and A+/B+/CDT+ (2%) types. Both types of C.difficile, A-/B-/CDT- and A+/B-/CDT-, which account for 77.5% of all isolates, were unable to produce the toxin (nontoxigenic). On the other hand, A+/B+/CDT+ and A+/B+/CDT- (22.5%), were able to produce toxin or were toxigenic.

**Conclusion:**

The frequency of *C. difficile* was about 21% and only 22.4% of *C. difficile* isolates were able to produce toxins. It is expected that *C. difficile* A+/B+/CDT± are toxigenic and related to *C. difficile* associated diarrhea (CDAD). Additionally, about 4.7% of hospitalized patients in ICU suffered from CDAD, which is higher than the rates reported from industrialized countries. Notably, 28% of isolates were *C. difficile* A+/B-/CDT- which only carries *tcdA* genes without toxin production.

## Introduction

*Clostridium difficile *(*C.difficile*), a rod-shaped gram-positive anaerobic spore forming bacterium, is part of the normal flora of 1-3% of healthy adults and 15-20% of infants. It is also the most important cause of CDAD (1). The spectrum of the CDAD ranges from mild diarrhea to pseudomembranous colitis (2). Many risk factors like age >60 years, duration of hospitalization, underlying diseases, gastric acid suppression and antibiotics exposure are associated with CDAD (3). Several broad-spectrum-antibacterial agents can induce CDAD, such as clindamycin, penicillins, sulfonamides/Trimethoprim (4), cephalosporins, aminoglycosides, macrolides and quinolones (5).

Prescribed antibiotics disrupt microbiota and promote colonization and overgrowth of *C. difficile*. Through production of toxins A and B, the bacterium induces bowel inflammation and ultimately causes diarrhea (3, 6). Some strains also produce actin-ADP-ribosylating toxin called binary toxin or *C. difficile* toxin (CDT). CDT positive strains are more commonly related to severe disease (7). The risk of CDAD in hospitalized ICU patients is about 20-25%, and 3-5% of them suffer from severe diarrhea. The mortality rate with fulminant CDAD in ICU patients can reach 34.7- 57% (8). 

Based on the type of toxin produced, *C. difficile *isolations fall into different baskets as follows: A^-^/B^-^/CDT, which is the non-toxin-producing type, while A^-^/B^-^/CDT^+^, A^-^/B^+^/CDT^+^, A^-^/B^+^/CDT^-^, A^+^/B^+^/CDT^-^, A^+^/B^+^/CDT^+^, A^+^/B^-^/CDT^+^(9) and A^+^/B^-^/CDT^-^ are considered as toxin production types (10). The prevalence of the strains in clinical samples is different (11). In most of the literature, CDAD is related to all strains, although there are some reports which suggest that *C. difficile* A^-^/B^-^/CDT^-^ might be normal flora and not associated with diarrhea (12). Additionally, there are limited investigations reported which indicate the role of *C. difficile *A^+^/B^-^/CDT^-^in human infectivity and its relation to CDAD (9).

Thus, the aim of the research was to determine the frequency of *C. difficile *toxin production types and CDAD in diarrhea hospitalized patients among Kerman ICU patients which could prove useful as there are few reports in this respect from Iran. 

## Methods

**Patients and samples**

During 2014-2015, 233 diarrheal stool samples were collected from ICU patients of Shahid Bahonar Hospital, Kerman, Iran. All patients with more than 3 bowel movements per day and antibiotic recipients were included in the study; patients who had not received antibiotics were excluded from the study. The stool samples were frozen at -20 ºC, for future investigations (13). Sufficient amounts of thawed samples, heated at 80 ºC for 10 min, were cultured on Clostridium difficile medium (MAST, UK) with 5% defibrinated sheep blood containing cycloserine (500 mg/L), cefoxitin (16 mg/L) and lysozyme (5mg/L). Cultured plates were incubated at 37 ºC in anaerobic jar (Anaercult, Germany) for 48-72 h and suspected colonies with particular odor, non-hemolytic and spore stain (sub-terminal spores) were considered as C. difficile (14). Isolates were considered as C. difficile after culture on brain heart infusion (BHI) blood agar (GIBCO, Scotland) for 72-96 h (good sporulation) and were stored in BHI broth with 40% glycerol at -70 ºC. Suspected isolates were confirmed by PCR based on 16s rRNA gene amplification as described later(15).

**DNA extraction**

Before DNA extraction, the isolates were removed from-70 ºC freezer and cultivated on BHI agar with 5% defibrinated sheep blood and incubated in anaerobic jar (Anaerocult,MERCK, Germany) for 24 h. Fresh colonies were used for DNA extraction (16). DNA extraction was performed using CinnaPure-DNA extraction kit for Gram positive bacteria (CinnaGen, Iran). Briefly, several colonies were selected and dissolved in 200 μL distilled water. The bacterial suspension was mixed for 5 s and centrifuged at 8000 rpm for 5 min (Labnet, USA). The pellet was dissolved in G prelysis buffer with 20 μL (500 μg/mL) lysozyme, incubated at 37 ºC for 45 min, and heated to 55 ºC. Then,10 μL ributinase was added to suspension and incubated at 55 ºC for 45 min. The suspension was used for DNA extraction according to the manufacturer's instructions kit and stored at -20 ºC for later use.

**PCR assay and electrophoresis**

PCR assay was performed according to the previous studies (15, 17). The C. difficile isolates were confirmed by 16S rDNA gene amplification. PCR was performed using master-mix (Amplicon, Denmark) by thermocycler (Biometra, Germany) and the amplicon was run in 1% agarose gel electrophoresis (Cleaver Scientific, UK) for 45 min. The gel was stained by green viewer stain and read by gel document (UVItec, UK).

**Toxin production evaluation**

In order to confirm toxin producing isolates, Clostridium difficile Toxins A&B ELISA Kit (tgcBIOMIC, Germany) was used which detects the toxins A and B in sample together (18). Briefly, about 106 CFU/mL fresh bacteria (24 h culture) were inoculated to the boiled BHI broth (GIBCO, Scotland) containing 0.05% L-cysteine (Merck, Germany) and 0.5% yeast extract (BBL, USA). After 48 h incubation in anaerobic jar, 1 mL of the culture was removed from the tube and centrifuged for 5 mins/10000 rpm(6). The supernatant was used to detect the toxin according to the manufacturer’s guidelines. 

## Results

**Frequency of** ***C. difficile*** **in diarrhea samples**

**Figure 1:**
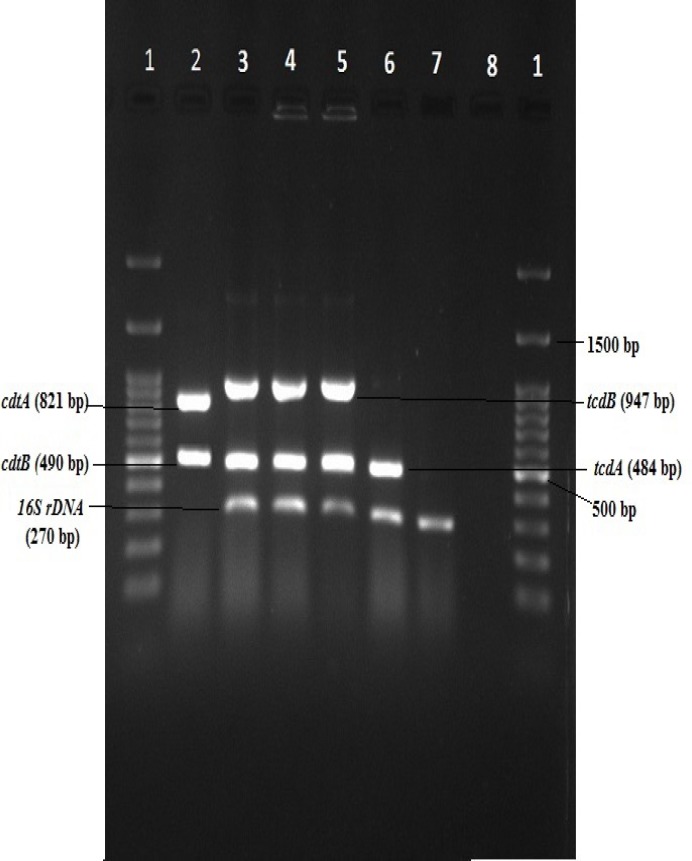
Gel electrophoresis of *16S rDNA*, toxin A, B and CDT genes. The PCR products were mixed and run to show toxin-producing isolates of *C.difficile*. Lane 1: DNA Ladder (100 bp), Lanes 2 & 3: *C. difficile* A^+^/B^+^/CDT^+^, Lane 4: *C. difficile* A^+^/B^+^/CDT^-^ (ATCC 9689), Lane 5: clinically isolated *C. difficile* A^+^/B^+^/CDT^-^ Line 6: *C. difficile* A^+^/B^-^/CDT^-^, Lane 7: *C. difficile* A^-^/B^-^/CDT, Lane 8: Negative Control

Totally, from 233 diarrheal samples, 49(21%) isolates were identified as *C. difficile*. Twenty-four isolates (49%) had no toxin genes and 25 (51%) were positive for toxin A (tcdA). Eleven isolates (24.5%) carried toxin B gene (tcdB) and one isolate was positive for Binary toxin (cdtA and cdtB) gene. On the other hand, 48.97% of all isolates did not carry any toxin genes (A-/B-/CDT-), and 28.57% were positive for only A+/B-/CDT-. However, the tcdA and tcdB genes (A+/B+/CDT-) and tcdA, tcdB and CDT genes (A+/B+/CDT+) were carried by 20.4% and 2.04% of total isolates respectively (Figure 1 & Table 1).

**Table 1:** Frequency of nontoxigenic and toxigenic *C. difficile* in diarrhea samples

*C.difficile*	toxin production type	No (%)
Nontoxigenic isolates* No (%)	A^-^/B^-^/CDT^-^	24 (48.97%)
	A^+^/B^-^/CDT^-^	14 (28.57%)
Toxigenic isolates* No (%)	A^+^/B^+^/CDT^-^	10(20.4%)
	A^+^/B^+^/CDT^+^	1 (2.04%)

* : According to toxin detection by ELISA technique which detects A and B toxins together.

**Frequency of toxigenic and nontoxigenic** ***C. difficile*** **isolates**

Evaluation of toxin production by the isolates revealed that both types of *C.difficile*, A-/B-/CDT- and A+/B-/CDT-, which account for 77.5% of all isolates, were unable to produce the toxin (nontoxigenic). On the other hand, the remaining isolates of C. difficile, A+/B+/CDT+ and A+/B+/CDT- (22.5%), were able to produce toxin or were toxigenic (Table 1).

## Discussion

In the current research, the frequency of *C. difficile *was about 21% among diarrheal samples from Shahid Bahonar hospital ICU. The results demonstrated that 49.57% of *C. difficile *isolates did not carry *tcdA*, *tcdB* and *CDT *(*cdtA *& *cdtB*) genes. About 20.4% of isolates carried *tcdA* and *tcdB* genes and one isolate (2%) was not only positive for *tcdA* and *tcdB *genes but also carried binary toxin genes (*cdtA, cdtB)*. Interestingly, 77.5% of the total isolates belonged to non-toxin production type or nontoxigenic (A^-^/B^-^/CDT^-^ and A^+^/B^-^/CDT^-^) and 22.5% were toxin producing or toxigenic (A^+^/B^+^/CDT^-^ and A^+^/B^+^/CDT^+^). Since toxigenic strains are associated with CDAD, it may be concluded that about 4.7% of ICU patients with diarrhea suffered from CDAD.

Although diarrhea is common in the ICU, about 20- 25% of these cases are related to infectious agents, of which* C. difficile* is the most important (8). The prevalence of *C. difficile *infectivity among the patients suffering from CDAD is different throughout the world. The worldwide prevalence of CDAD is 0.9% and 2% in the general population and ICU patients, respectively (19). A similar pattern is observed in Europe (1%) and Asia (3%) (19).Additionally, investigations have revealed that 3.6%, 3.3%, 3.3%, 0.9%, 2.4% and 20% of CDAD in ICU hospitalized patients of the USA, Canada, the UK, France, China and Taiwan are related to *C. difficile* infectivity, respectively (2, 20, 21). The prevalence of *C. difficile* infectivity and CDAD has been less studied in Iran, especially in ICU hospitalized patients. In previous Iranian studies, the prevalence of *C. difficile *and CDAD has been studied in other parts of the hospital. These studies have shown that the prevalence of CDAD was about 6.1- 20% and 5.3% in hospitalized patients and those with gastrointestinal complaints, respectively (22-24). The current investigation exclusively addresses the epidemiology of *C. difficile* in ICU patients. Based on the results and toxin positive strain which is more often related to CDAD, the prevalence of CDAD among ICU patients (4.7%) is relatively higher than other regions of the world and it seems that *C. difficile* can be considered as the main cause of CDAD among ICU hospitalized patients in Kerman, Iran.

Toxin production type A^-^/B^-^/CDT^- ^was the most prevalent type observed in our study. Similar to our results, several studies have shown that the A^-^/B^-^/CDT^- ^toxin production type is the most prevalent (42- 50%) in clinical data (25, 26). In some studies, they have been regarded as pathogenic while as non-pathogenic in others (25, 27). In this study, they are considered as nonpathogenic.

Another toxin production type, A^+^/B^+^/CDT^- ^which is clearly associated with CDAD, has up to 71.6% prevalence among *C. difficile* toxin production types globally and 100% in Iran (22, 28). This strain was prevalent in our isolates, although its prevalence was lower than other studies in Iran and other parts of the world.

Additionally, the results revealed that the prevalence of *C. difficile *A^+^/B^-^/CDT^- ^was 28.57% among the ICU hospitalized patients with diarrhea. This toxin production type has also been detected in Iran. Goudarzi *et al.* reported that 6.7% of *C. difficile* related CDAD belonged to this type (11). The actual role of these isolates in inducing CDAD has not been established. They have been rarely reported by investigators (29, 30). Rupnik believes that this is due to the wrong choice of primer that may amplify the reaming *tcdA* gene in Pathogenicity Locus(9). On the other hand, Monte and colleagues have isolated this toxin production type from clinical samples and it was found to be associated with CDAD (31). In this study, A^+^/B^-^/CDT^-^ isolates were positive for the presence of *tcdA* gene. The toxins (A and B by ELISA kit which detects the two toxins together) were not detected in the medium by commercial ELISA kit. It means that A^+^/B^-^/CDT^-^ isolates do not express *tcdA* gene or maybe the level of toxin is too low to be detected by ELISA methods.

The A^+^/B^+^/CDT^+ ^Toxin production types are able to produce a third toxin which is named CDT. The number of these isolates in clinical samples is growing and has increased from 0% to 45% in the past three decades (32-34). CDT has been reported in Iran and its prevalence in clinical samples has reached to 32% (22). Therefore, the prevalence of A^+^/B^+^/CDT^+^ type was low in this study (2%) in comparison to other reports from Iran and other geographical regions in the world.

In total, non-toxigenic isolates are prevalent in clinical samples. About 28% of isolates carry only *tcdA* gene without toxin A production and their role in CDAD is not clear. The prevalence of A+/B+/CDT- and CDT positive toxin production types is low in comparison to the global prevalence, but it is still the leading cause of CDAD among ICU patients. Totally, 22.4% of *C. difficile *isolates carry *tcd A *and *tcdB* genes. Therefore, about 4.7%of total diarrheal patients hospitalized in ICU suffer from CDAD which is higher than other geographical regions in the world such as industrialized countries.
